# Insight into the Peopling of Mainland Southeast Asia from Thai Population Genetic Structure

**DOI:** 10.1371/journal.pone.0079522

**Published:** 2013-11-04

**Authors:** Pongsakorn Wangkumhang, Philip James Shaw, Kridsadakorn Chaichoompu, Chumpol Ngamphiw, Anunchai Assawamakin, Manit Nuinoon, Orapan Sripichai, Saovaros Svasti, Suthat Fucharoen, Verayuth Praphanphoj, Sissades Tongsima

**Affiliations:** 1 National Center for Genetic Engineering and Biotechnology (BioTeC), Khlong Luang, Pathum Thani, Thailand; 2 Inter-Department Program of Biomedical Sciences, Chulalongkorn University, Pathumwan, Bangkok, Thailand; 3 Faculty of Pharmacy, Mahidol University, Rajathevi, Bangkok, Thailand; 4 School of Allied Health Sciences and Public Health, Walailak University, Thai Buri, Nakhon Sri Thammarat, Thailand; 5 Thalassemia Research Center, Mahidol University, Salaya, Nakhon Pathom, Thailand; 6 Center for Medical Genetics Research, Rajanukul Institute, Dindaeng, Bangkok, Thailand; University of Florence, Italy

## Abstract

There is considerable ethno-linguistic and genetic variation among human populations in Asia, although tracing the origins of this diversity is complicated by migration events. Thailand is at the center of Mainland Southeast Asia (MSEA), a region within Asia that has not been extensively studied. Genetic substructure may exist in the Thai population, since waves of migration from southern China throughout its recent history may have contributed to substantial gene flow. Autosomal SNP data were collated for 438,503 markers from 992 Thai individuals. Using the available self-reported regional origin, four Thai subpopulations genetically distinct from each other and from other Asian populations were resolved by Neighbor-Joining analysis using a 41,569 marker subset. Using an independent Principal Components-based unsupervised clustering approach, four major MSEA subpopulations were resolved in which regional bias was apparent. A major ancestry component was common to these MSEA subpopulations and distinguishes them from other Asian subpopulations. On the other hand, these MSEA subpopulations were admixed with other ancestries, in particular one shared with Chinese. Subpopulation clustering using only Thai individuals and the complete marker set resolved four subpopulations, which are distributed differently across Thailand. A Sino-Thai subpopulation was concentrated in the Central region of Thailand, although this constituted a minority in an otherwise diverse region. Among the most highly differentiated markers which distinguish the Thai subpopulations, several map to regions known to affect phenotypic traits such as skin pigmentation and susceptibility to common diseases. The subpopulation patterns elucidated have important implications for evolutionary and medical genetics. The subpopulation structure within Thailand may reflect the contributions of different migrants throughout the history of MSEA. The information will also be important for genetic association studies to account for population-structure confounding effects.

## Introduction

The human population genetic history of Asia is complex, which is highlighted by the controversy surrounding the earliest migrations through Asia. One school of thought is that Asians are descended from two major ancestral groups, the earliest who migrated via a southern coastal route and a later group who spread across northern and eastern Asia [1]. An alternative hypothesis from genome-wide surveying of genetic variation across 73 Asian populations is that there was only one major migration pattern, in which East Asian peoples are descended from southern migrants who migrated north [2]. The controversy has been reignited following analysis of ancient human genomes from Central Asia [3] and Australia [4] which tend to support the two-wave hypothesis. The great diversity across Asia shaped by multiple migrations and population expansions throughout history will only be realized by more in-depth population genetic studies [5]. This gap in knowledge has begun to be addressed by large-scale studies of Asian populations sampling thousands of individuals, which have revealed stratification (distinct subpopulations) among the populations of India [6], Japan [7], and China [8,9]. The degree of genetic stratification in these populations largely reflects known ethno/cultural/linguistic divisions and patterns of assumed ancestry. 

Thailand lies at the heart of mainland Southeast Asia (MSEA), the region in which peoples speaking Tai-Kadai, Austroasiatic (Mon-Khmer), Sino-Tibetan, Hmong-Mien and Austronesian languages are present. The contemporary populations of this region are dominated by Tai language speakers (Thai and Laotian) and Austroasiatic speakers (Cambodian and Vietnamese). Most importantly, Thailand is located at the crossroads of ancient human migration paths between North and East Asia and Island Southeast Asia. Therefore, the genetic footprints of ancestral migrants may be present among people in this region. The earliest archaeological evidence of humans in MSEA was obtained in southern Thailand, dating to approximately 25,000 Years Before Present (YBP) [10], which is among the oldest remains documented in Southeast Asia [11]. mtDNA analysis of this specimen showed close relationship with the present-day Semang population in Peninsula Malaysia [12]. The Semang are an aboriginal “Negrito” people (distinguished by their darker skin pigmentation, different hair morphology, and short average stature), who may have been living continuously in Southeast Asia since the earliest Asian migration to Australia 60-75,000 YBP [13]; other Negrito populations elsewhere in Southeast Asia have a similarly ancient origin [14,15]. The southern part of Thailand was thus first populated by “Australo-Melanesian” [13] ancestral people. On the other hand, it is not clear how extensively populated MSEA was at this time, since archaeological evidence for communities and settlement prior to the Bronze Age (approximately 4500 YBP) in MSEA is sparse [16]. Bellwood (1993) argued that the earliest humans in MSEA would have been restricted to the coastal regions and not penetrated inland as the environment was not suitable for a foraging lifestyle [17]. Therefore, it is likely that the earliest populations of significance in MSEA were established by Austric agriculturalist people, the ancestors of Austroasiatic and Austronesians, who may have originated in Southern China. These migrants spread along river basins in MSEA reaching the Malaysian Peninsula in the Neolithic period [16]. Mitochondrial DNA study of Bronze and Iron age human remains from central Thailand was concordant with the presence of autochthonous Austric people in central Thailand [18]. Tai people migrated from southern China into northern Thailand more recently, establishing settlements in Thailand alongside the autochthonous Austrics. Eventually, the Tai became dominant, establishing control over northern Thailand from the 8^th^ Century AD [19]. Later Tai domination covering much of present-day Thailand was evidenced by the Sukhothai dynasty (established 13^th^ Century AD) and the Ayutthaya dynasty (established 15^th^ Century AD), although the southern region of Thailand was essentially autonomous and ruled by Malay vassals until the 19^th^ Century AD. During this most recent phase of Thai history, a large influx of migrants from southern China occurred [20]. Within the same period, other MSEA populations also experienced similar patterns of immigration and assimilation of southern Chinese, with Chinese influence greatest in Vietnam [21]. 

Despite the strategic location of Thailand in MSEA, there has been no large-scale study of its population’s genetic variation. Previous studies of human genetic diversity in Thailand were done with limited marker sets [22,23], and/or limited sampling (restricted to ethnic minorities); [2,22,24-28]. To better our understanding of mainland Southeast Asian and Thai population genetics, we undertook a study of Thai population genetic structure. The Thai population dataset comprises 992 individuals genotyped for 552,386 autosomal SNP markers. We found that the Thai population is genetically distinct from other Asian populations, but there is evidence of shared ancestry supporting the known origins and historical migration patterns across MSEA. Four Thai subpopulations were resolved which are distributed differently across Thailand. Interestingly, the most highly differentiated markers which can distinguish the four Thai subpopulations include several within genes which are known to affect traits such as skin pigmentation and susceptibility to common diseases. 

## Methods

### Ethical statement

The recruitment of human subjects was approved by the ethical review committee for research in human subjects (Mental Health and Psychiatry): Ministry of Public Health, Thailand (CCA No. Si 32/2009). 

Three SNP genotyping datasets were analyzed in this study. The first dataset is from a worldwide population study of 850 individuals from 40 populations published in [29]. The genotypic data from this dataset were obtained using the Affymetrix Human SNP Array 6.0 comprising 246,554 SNPs that passed quality control (after removal of markers that deviate from Hardy-Weinberg Equilibrium (HWE) (*P*< 5.5×10^-8^) and missing data >10%). The second dataset is a case-control association study to identify genetic factors of major depressive disorder. Human subjects for genotyping were recruited according to the ethical statement mentioned above. The dataset comprises 374 individuals (186 cases and 188 controls) collected from North, Northeastern, Central and Southern regions of Thailand. The DNA samples were genotyped using the Illumina Human 610-Quad BeadChips Array at RIKEN, Japan. The total number of genotyped SNPs is 593,542. SNPs were filtered to remove markers in high LD (linkage disequilibrium r^2^ > 0.5), high deviation from HWE (*P*<10^-3^) and missing data >5% using the PLINK tool. After filtering, 438,503 SNP markers remained for further analyses. Disease association test was performed using the PLINK tool. No marker passed the threshold for Bonferroni-corrected significance (*P*<10^-7^). The top 50 ranked markers are shown in Table S1. The third dataset is a case-control study to identify modifying genetic factors that cause patients with β^0^-thalassemia/hemoglobin E with different spectrums of disease severities. The study collected 383 severe patients and 235 mild patients and performed case-control association. The data and association study were previously published in [30]. Genotyping was done using the same platform as with the second dataset, i.e. 610-Quad BeadChips Array for a total number of 593,542 SNPs. Note that both datasets 2 and 3 were from two *independent* case-control association studies of Thais where individuals’ samples were collected from different regions in Thailand by different Principal Investigators. For datasets 2 and 3, individuals were asked to assign a geographical label for themselves (North, South, Northeast or Central) based on their place of birth, or their parent’s place of birth. We tested for systematic differences of allele frequency caused by sampling bias between datasets 2 and 3 for 438,503 SNPs. A Bonferroni corrected P-value of 10-7 was used as the significance threshold. In accordance with PLoS policy on data availability, requests to access datasets 2 and 3 should be sent to Dr. Verayuth Prapanpoj and Prof. Suthat Fucharoen, respectively.

### Population analyses

The analyses were done in two stages. First we observed the relationship between Thais and other related populations. The common polymorphic SNPs from all three datasets (41,569 SNPs) were used for population structure analysis. This marker set includes only SNPs that have the same reference SNP identification code (rs-id) between the Affymetrix and Illumina SNP array platforms. For some of these SNPs in common, the SNP calling on one platform is the complement of the other platform, i.e., A/G versus T/C. In these cases, the Affymetrix SNP calls were complemented to be the same as Illumina’s. Common SNPs in which the base identity of the variant SNP was ambiguous on either platform were excluded. Finally to ensure that no hidden technical bias may exist between the two platforms for the common marker set, minor allele frequencies (MAF) for each SNP were calculated from a control population with 136 samples from Affymetrix [29] and 1,182 samples from Illumina [31] platforms, respectively. The scatter plot and the calculated correlation coefficient of MAFs do not show any evidence of biased MAFs (Figure S1).

Population structure was analyzed first by bootstrapping neighbor-joining (NJ) tree of the three combined datasets (1,842 individuals genotyped for 41,569 markers common among the two genotyping platforms) using the *seqboot*, *gendist*, *consense* and *neighbor* programs within the PHYLIP program suite (with default parameters) [32]. Allele frequencies of each population were calculated using *seqboot* (individuals with the same label were assumed to belong to the same population). The dissimilarity matrix was calculated from the matrix of allele frequencies using the *gendist* program. The *neighbor* module was used to construct NJ-trees from these matrices. Finally, *consense* was used to generate the consensus tree with bootstrapping values using the Pygmy population as an out-group. The unrooted phylogram was plotted using Dendroscope [33]. 

The ipPCA program [34,35] was used with stopping criterion EigenDev=0.21 [35] to assign 1,842 individuals genotyped for 41,569 markers into subpopulations in an unsupervised manner disregarding the population labels for each individual. The data matrices were generated with each row representing a SNP profile for an individual and each column representing a SNP genotype (0: homozygous wild type, 1: heterozygous and 2: homozygous variant). The ADMIXTURE [36] program was used to estimate individual ancestries of each individual from the same SNP genotypic data from K=2 to K=10 ancestors. ADMIXTURE uses the same maximum likelihood principle of STRUCTURE [37] to infer the ratio of assumed ancestors for each individual. The admixture ratios of individuals were plotted using the ‘bar’ function in MATLAB version 2009b on Linux operating system. 

High-resolution study of population substructure within the Thai population was performed on the combined datasets 2 and 3 (992 individuals genotyped for 438,503 SNPs). Subpopulations were assigned using ipPCA with stopping criterion EigenDev=0.21. ADMIXTURE was used to estimate individual ancestries from K=2 to K=4 ancestors. Genome-wide Fst values [37] were calculated among all pair-wise combination of ipPCA assigned subpopulations using the Arlequin software with default settings [38], and the significance tested by permutation testing option for 1023 permutations. Fst values for each of the 438,503 SNPs among all pair-wise combination of ipPCA assigned subpopulations were calculated using the Arlequin software. The SNPs were then ranked according to Fst values in all pairwise subpopulation comparisons.

## Results

**Figure 1:**
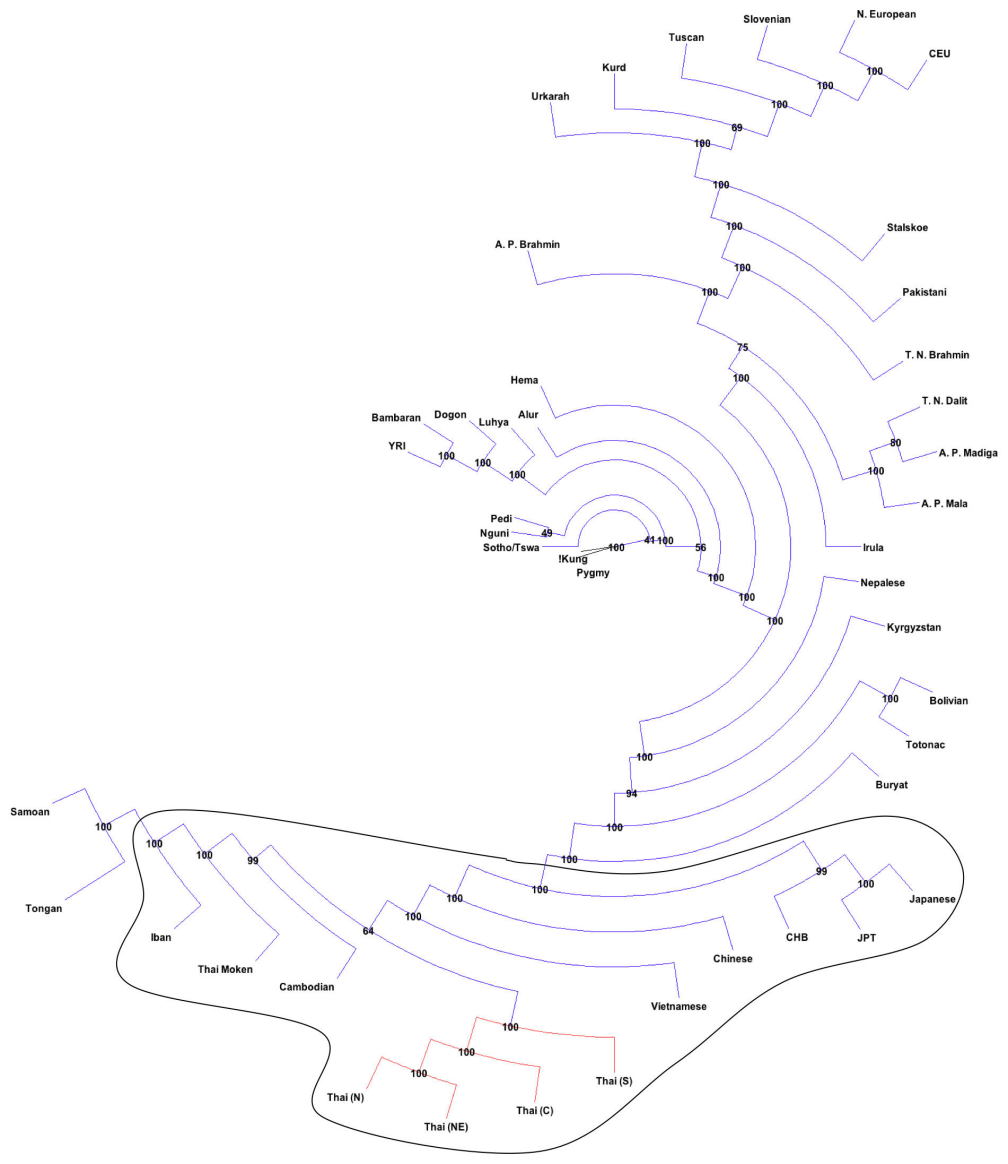
Consensus population Neighbor-Joining unrooted Tree. An amalgamated worldwide dataset of 1842 individuals genotyped for 41,569 SNPs was analyzed by PHYLIP. The minor allele frequencies for each population were calculated and used as input to produce the dissimilarity matrix using Nei’s approach for unrooted NJ tree. The data were comprised of 850 individuals from 40 populations (dataset no.1; [29]), 618 Thai individuals (dataset no. 2; [30]) and 374 Thai individuals (dataset no. 3; this study). The Thai individuals from datasets no. 2 and 3 were assumed to belong to the same population and then separated into regional subpopulations based on self-reported origins: Thai (C), Thai (NE), Thai (N) and Thai (S). The other population labels are the same as those reported previously in [29], except “Thai” which has been re-labeled as “Thai-Moken”. The consensus tree from 100 bootstrap replicates is shown, and the bootstrap values are indicated on each node of the tree. Southeast and East Asian populations are ringed and the clades separating Thai subpopulations are in red.

In order to frame the Thai population in a worldwide context, the Thai genetic data were combined with the worldwide population data published in [29]. The combined dataset of 1,842 individuals was analyzed using the 41,569 SNP markers common to the two different microarray platforms (File S1). Consensus neighbor-joining (NJ) unrooted tree of populations assigned using the ethno-geographical information (Figure 1) reveals that the Southeast and East Asian populations are distinct from the rest of the world. Moreover, all the Southeast and East Asian populations occupy *distinctive* positions (clades with 100% bootstrap support) from other populations except for Thai Moken and Cambodian people who occupy positions in the tree with weaker bootstrap support. It is striking that the Thai subpopulations (according to the regional geographic origins) are also distinct.

**Figure 2:**
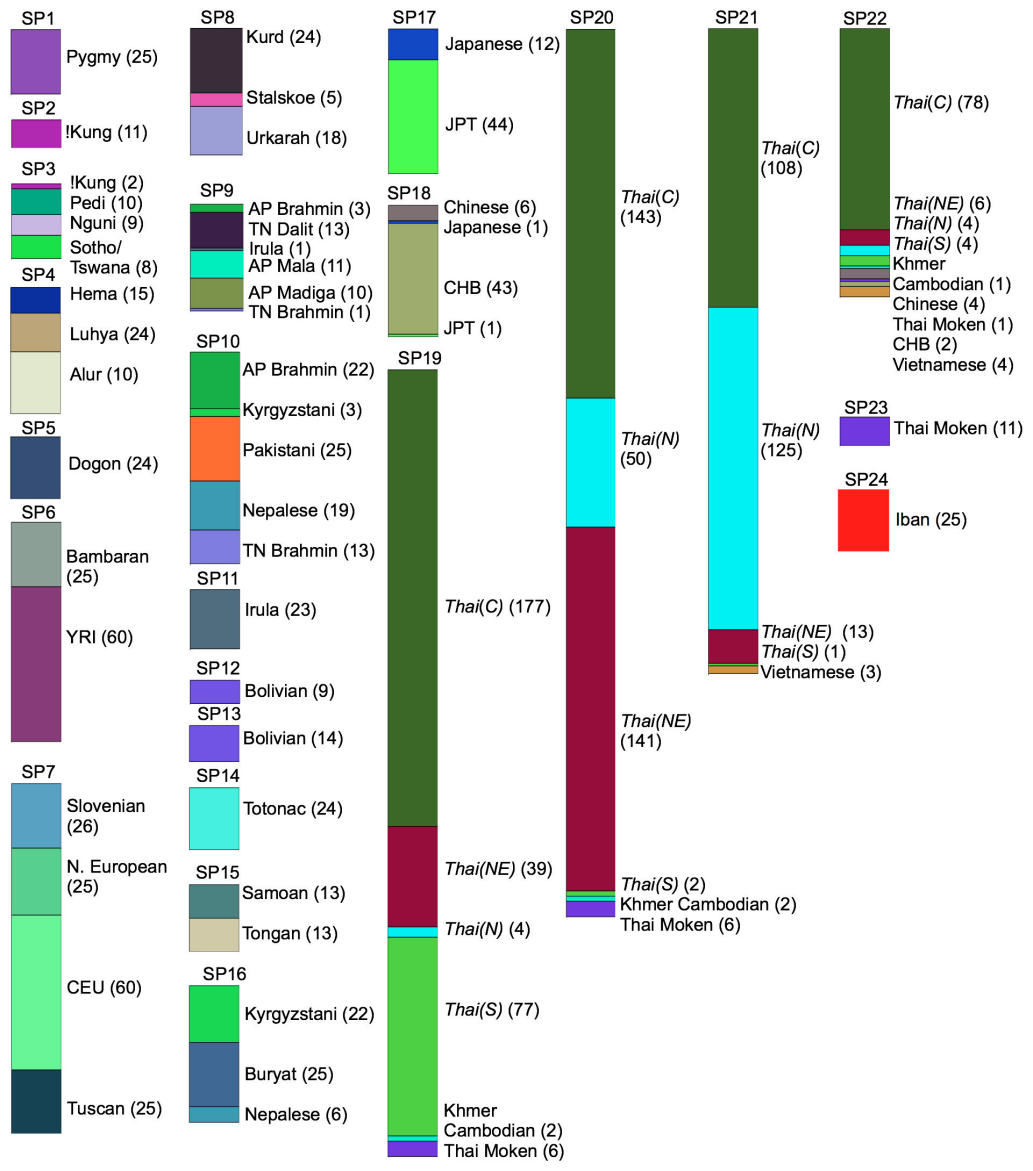
ipPCA subpopulation assignment. The amalgamated worldwide dataset of 1842 individuals was analyzed by ipPCA. The Thai ethno/geographical labels pertaining to datasets 2 and 3 are italicized; all other labels are the same as those shown in Figure 1. Individuals were assigned into 24 genetically distinct subpopulations (SP1 to 24) by ipPCA. 20 Thai individuals that could not be assigned to subpopulations are not shown. The height of each subpopulation bar is proportional to the number of assigned individuals.

Next, subpopulation genetic structure was analyzed using the ipPCA algorithm [34,35]. Subpopulation assignment of individuals by this algorithm is performed using an unsupervised clustering approach that does not use the individuals’ ethno-geographical information. The subpopulations resolved by this algorithm are genetically homogeneous with no significant variation from that expected for a random collection of unrelated individuals. The resulting 24 subpopulations assigned by ipPCA generally reflected the individual ethno-geographical labels in agreement with the pattern from the consensus NJ tree (Figure 2), but with some interesting discrepancies. Mainland Thais were assigned to four subpopulations (SP19-22) together with some of the Thai Moken individuals from Xing’s dataset. However, Thai Mokens were assigned exclusively to SP23. Interestingly, all Vietnamese individuals were assigned with Thais in SP21 and SP22 and all Cambodians were assigned with Thais in SP19, 20 and 22. Some Chinese individuals were also assigned to SP22 with Thais, Vietnamese and Cambodians. Another important observation is that among the predominantly Thai subpopulations SP19-22, there appears to be regional bias. For instance, SP19 contained the majority of Southern Thais, while SP20 contained the majority of Northeastern Thais and SP21 the majority of Northern Thais. SP22 is dominated by Central Thais, although this subpopulation constitutes only a minority of the total of Central Thais. 20 Thai individuals appeared as genetically distinct “outliers” that could not be assigned to a specific subpopulation and were separated by ipPCA at different iterations of the algorithm (see Figure S2). 

**Figure 3:**
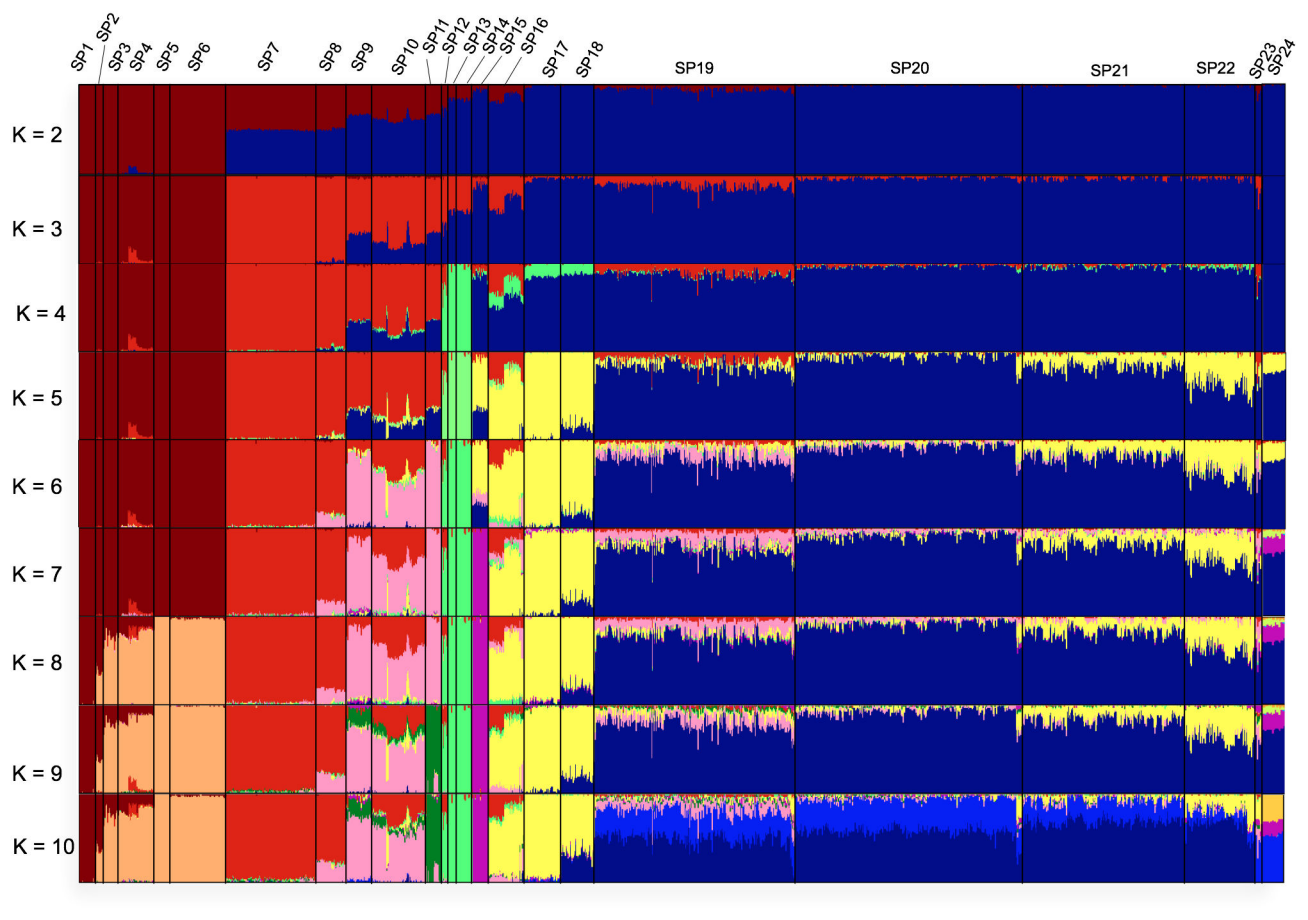
Ancestry analysis by ADMIXTURE. The amalgamated worldwide dataset of 1842 individuals was analyzed by the ADMIXTURE program. The number of K ancestral clusters was varied from 2 to 10. Individuals were grouped according to the subpopulation assignments made by ipPCA (Figure 2). The ordering of individuals within each subpopulation group is arbitrary.

Next, admixture ratios of inferred ancestry (K=2 to 10) for each individual (ipPCA outliers excluded) were determined using the ADMIXTURE program [36]. When individuals are grouped according to their subpopulation assignments made by ipPCA, subpopulation-distinctive admixture patterns were observed at K=7 (Figure 3). Analysis with higher K ancestral clusters was not much more informative, since no new major ancestral components of any subpopulation were apparent. SP19-22 containing mostly Thai individuals were assigned with one major ancestral component (blue) and two minor components (pink and yellow) at K=7. The major blue component is also a major component of SP24 (Iban individuals) and to a lesser extent SP18 (mostly Chinese individuals). 

**Figure 4:**
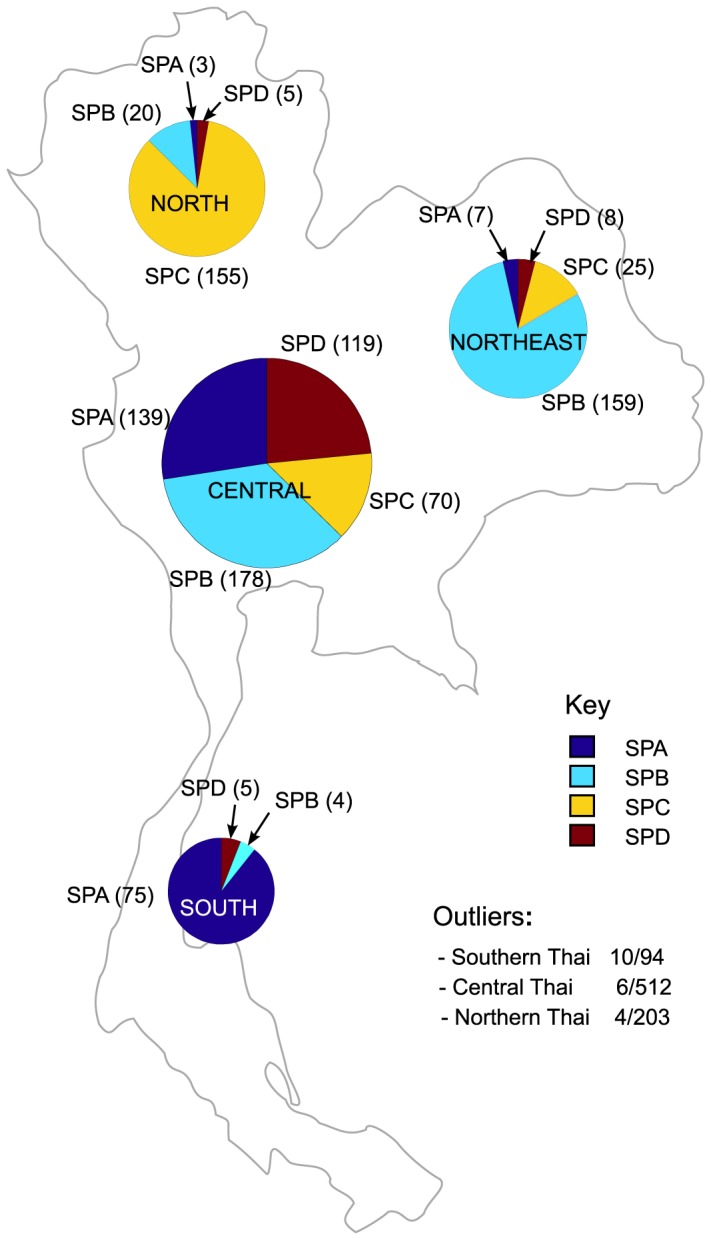
High-resolution ipPCA assignment of 992 Thai individuals. 992 Thai individuals from datasets no. 2 and 3 were combined and analyzed by ipPCA utilizing 438,503 SNP markers. Four subpopulations (SPA, B, C and D) were resolved by ipPCA, whereas 20 individuals could not be assigned to a subpopulation and are separated as “Outliers”. The proportions of individuals assigned to each subpopulation are shown for each geographical region based on the available information of self-reported origin (North, Northeast, Central, and South).

**Figure 5:**
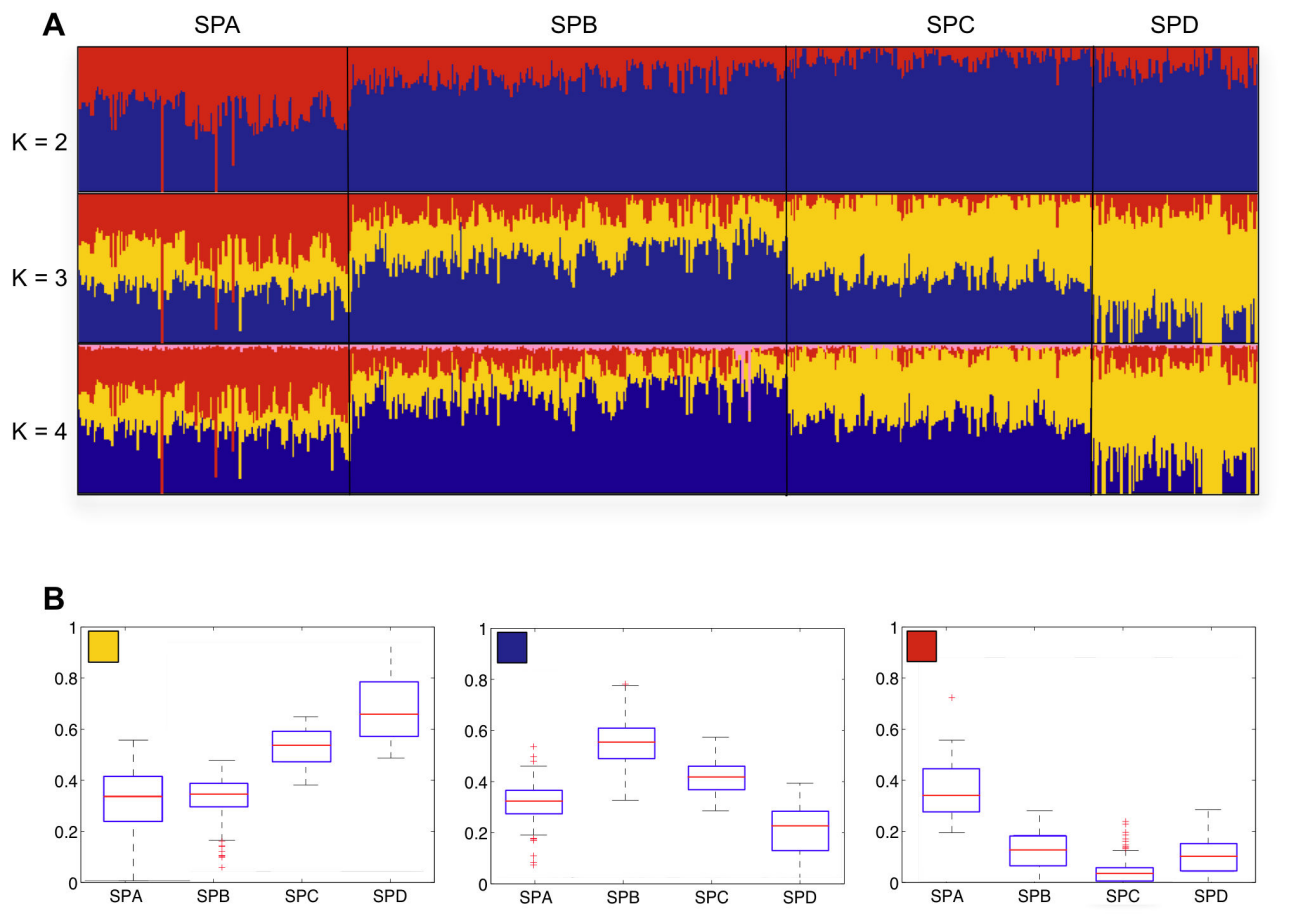
High-resolution ancestry analysis of 972 Thais. A) 972 individuals from datasets no. 2 and 3 (ipPCA Outliers removed) were combined and analyzed by ADMIXTURE utilizing 438,503 SNP markers. The individuals were grouped according to the subpopulation assignments made by ipPCA shown in Figure 4. B) Box and whiskers plots for K=3 ADMIXTURE-inferred ancestral components (blue, yellow and red) of ipPCA-assigned subpopulations SPA, B, C and D.

Next, having shown substructure among the mainland Thai population with relatively few markers, a higher resolution analysis of 992 Thai individuals was performed using 438,503 SNP markers. Subpopulation assignment by ipPCA revealed four subpopulations labeled SPA, B, C and D (Figure 4). 20 outlier individuals could not be assigned to these four subpopulations (Figure S3), and were excluded from further analysis. The assignment of individuals to the four subpopulations SPA, B, C and D was correspondent with SP19, 20, 21, and 22, respectively from low-resolution ipPCA (Figure 2), with minor discrepancies (Table S2). Regional bias in subpopulation assignment was apparent, with predominance of South individuals in SPA, Northeast individuals in SP-B, and North individuals in SPC. SPD contains predominantly Central individuals, although this subpopulation does not constitute the majority of Central individuals. The level of variance in allele frequency among subpopulations SPA, B, C and D was determined by Fst analysis, and all pairwise comparisons were significant as shown by permutation testing (Table 1). Therefore, the population substructure found by ipPCA was cross-validated by Fst analysis. An alternative explanation for the substructure among the Thai samples is that the patterns reflect the individual’s disease status or an artifact of the sample collection rather than general population structure. To test this hypothesis, deviation of minor allele frequency of the Thalassemia dataset was compared with the Major depressive disorder dataset from the expected ratio for all markers (438,503) by chi-squared analysis. No markers showed significant deviation (Table S3), indicating that the amalgamation of two datasets carried no bias for population structure analysis. Admixture analysis of these individuals with 438,503 SNP markers shows that each subpopulation has distinct patterns of admixture ratios at K=3; the fourth ancestral component is not informative as it carries only a tiny proportion of the ancestry in almost all individuals (Figure 5). 

**Table 1:** Pairwise Fst analysis of Thai subpopulations.

	SP-A	SP-B	SP-C	SP-D		
SP-A	0	0.0020*	0.0032*	0.0034*		
SP-B		0	0.0015*	0.0025*		
SP-C			0	0.0023*		
SP-D				0		

* Significance tests were performed with 1023 permutations and their resulting P-value < 0.01

Having demonstrated substructure among the Thai population, an investigation of the genomic regions most diverged among the subpopulations was performed. The markers were ranked according to their Fst values in pairwise subpopulation comparisons (Table S4). Among the top-ranked markers with highest Fst between subpopulations, several were present in genes, and a few have been reported previously to affect phenotypic traits such as skin pigmentation and susceptibility to disease in other populations (Table 2). SPA is distinguished by high frequencies of SNPs in the OCA2 and SLC24A5 genes, and these markers are strongly associated across different populations with skin pigmentation [38]. The same markers are present at lowest frequency in SPC compared with SPA, B and D. SPB is distinguished by high frequency of the rs987870 SNP, which present in the HLA-DPB1 gene and is associated with pediatric asthma in different Asian populations [39]. SPD is distinguished by high frequency of several SNPs previously reported to be associated with disease in East Asian populations, including SNPs in the ADH4, ALDH2, BRAP and PANK4 genes which are associated with upper aerodigestive tract cancer, metabolic effect of alcohol, metabolic syndrome and type 2 diabetes, respectively [40-43]. Although some of the markers that distinguish the Thai subpopulations have phenotypic associations in other populations, phenotypic associations for the majority of distinguishing markers have not been reported.

**Table 2:** Top-ranked SNPs with highest Fst between subpopulations with known phenotypic association.

												**Subpopulation minor allele frequencies**				
**Fst^a^ value (SPx-SPy)**	**Rank^b^**	**rsID**	**Chr**	**Position**	**Allele**	**Region**		**Gene**		**Reported Phenotypic association**		**SPA**	**SPB**	**SPC**	**SPD**		
0.023 (SPA-SPB)	109	rs4778220	15	25872900	T/G	intron		OCA2		hair color and skin pigmentation		0.1	0.03	0.02	0.02		
0.046 (SPA-SPC)	12	rs1426654	15	46213776	AG	coding		SLC24A5		skin pigmentation		0.14	0.04	0.02	0.04		
0.021 (SPB-SPC)	29	rs987870	6	33150858	T/C	Flanking 5’UTR		HLA-DPB1		pediatric asthma		0.16	0.24	0.13	0.12		
0.037 (SPA-SPD)	65	rs3805322	4	1E+08	A/G	intron		ADH4		upper aerodigestive tract cancer		0.17	0.19	0.21	0.35		
0.042 (SPB-SPD)	6	rs671	12	1.11E+08	T/C	coding		ALDH2		metabolic effect of alcohol		0.1	0.06	0.06	0.19
0.041 (SPB-SPD)	16	rs3782886	12	1.11E+08	A/G	coding		BRAP		metabolic syndrome		0.1	0.06	0.06	0.18
0.038 (SPB-SPD)	22	rs7535528	1	2434274	T/C	coding		PANK4		type II diabetes		0.22	0.18	0.21	0.36
0.046 (SPA-SPC)	13	rs2517646	6	30230554	T/C	intron		TRIM10		highly differentiated SNP between Chinese subpopulations		0.23	0.12	0.08	0.1
0.045 (SPA-SPC)	17	rs11130248	3	50327204	A/G	Flanking 5’UTR		COL4A1		susceptibility loci for keloid in the Japanese population		0.21	0.12	0.07	0.12
0.044 (SPA-SPC)	20	rs2291652	3	1.97E+08	T/C	coding		MUC3		endometriosis-related infertility		0.27	0.16	0.11	0.19
0.048 (SPA-SPD)	20	rs1165153	6	25925768	T/C	intron		SLC17A1		development of gout		0.38	0.36	0.27	0.18
0.035 (SPB-SPD)	33	rs103294	19	59489660	T/C	Flanking 3’UTR		LILRA3		prostate cancer		0.27	0.15	0.17	0.31

^a^   Fst is the value between the specified pair-wise subpopulation comparison shown in parenthesis.

^b^ Rank value refers to the rank of Fst value for the same pair-wise subpopulation comparison (see Table S4 for complete ranked list)

## Discussion

In this study, we have attempted to fill an important gap in the knowledge about human population genetics in MSEA. Consensus NJ tree (Figure 1) and ipPCA subpopulation assignment using a limited marker set (Figure 2) showed that genetically distinct groups exist among Eurasian peoples that are broadly aligned with ethno-linguistic labels. Among these populations though, there were some unexpected patterns. Five subpopulations of Thais were clearly distinct by NJ tree and ipPCA assignment, including a subpopulation of Thai individuals from the Xing dataset (SP23, Figure 2). The Thai individuals in SP23 were sampled from the Moken minority ethnic group, who are distinct from majority Thais in that they have lived continuously in coastal areas of Southern Thailand for several generations and speak their own Austronesian language [29]. The distinct ethnic identity of the Moken may thus have acted as a barrier to gene flow and led to genetic divergence from the majority of Thai people. The existence of the other four Thai subpopulations was unexpected as there are no ethno/linguistic distinguishing labels among these individuals. Geographical origin could partly explain the divergence of these subpopulations, with South, North and Northeastern Thais predominating SP19, 20 and 21 respectively. Central individuals comprised the majority of SP22, but this subpopulation was only a minority of the total of Central individuals. Also surprising was the genetic similarity of other MSEA peoples with Thais, i.e., Cambodians were assigned with Thais in SP19, 20 and 22, while Vietnamese were assigned with Thais in SP21 and SP22 (with some Chinese also). Although the sampling of Cambodians and Vietnamese was much lower than Thais, the patterns suggest that the subpopulation structure within Thailand is representative of MSEA. 

From the Admixture analysis at K=7, MSEA people in SP19-22 were shown to be represented by one major ancestral component (Figure 3). This component could represent the ancestry of autochthonous Austroasiatic people present in MSEA before the Tai expansion (see Introduction). This ancestry is also a major component of SP24 which is comprised of Austronesian-speaking Iban from the Peninsula Malaysia. Previous genetic analysis of Iban showed close association with MSEA people, suggesting that the ancestors of Iban were from MSEA [44]. The MSEA ancestors of the Iban and other Austronesians in MSEA were probably Austric-speaking migrants who migrated from central Thailand to the Malaysian Peninsula [45]. The most common mtDNA haplotypes in the Austronesian-speaking Thai Moken are also found in aboriginal peoples of the Malaysian Peninsula [46], and these Malay aborigines speak Austronesian and Austroasiatic languages. Among other Austronesian-speaking minorities in MSEA, the Cham group in Vietnam also has a closer genetic affiliation with Austroasiatic populations in MSEA than with Austronesian populations from Island Southeast Asia [47].

Four genetically distinct Thai subpopulations were assigned using 438,503 SNPs with essentially the same assignment as with the smaller marker set. The minor discrepancy between the two ipPCA analyses performed with different numbers of markers is clustering error since the ability to resolve population structure is dependent on the number of markers available [48]. Even with a larger marker set, a small number of Thai individuals could not be assigned to subpopulations by ipPCA and instead separated as outliers at various clustering steps of ipPCA (Figures S2 and S3). These outlier individuals may constitute individuals with recent non-SE Asian ancestry, or unaccounted for familial relationship. Such outlier individuals are likely to be present in any large population study and are typically excluded [49,50]. Among the four geographical regions of Thailand, the Central region is the most diverse in that no one subpopulation is dominant. In contrast, the other regions are more genetically homogeneous. The high diversity of the Central region is likely because of recent migration, as this region has been the economic center of the country since the 15^th^ Century AD Ayutthaya period. Although SP22/SPD constitutes a minority of Central Thais, SP22/SPD individuals are concentrated in this region. Several Chinese, Vietnamese and a Cambodian individual were assigned by ipPCA with Thais in SP22. One explanation for this pattern, given the modern history of MSEA is that the Thais, Vietnamese and Cambodian in SP-22 may be descendants of recent Chinese migrants. In support of this conjecture, Admixture analysis showed that these individuals share a prominent ancestry with predominantly Chinese SP18 individuals (yellow component in Figure 3). Moreover, among the top-ranked SNP markers which are present at high frequency in SP-D and distinguish it from the other four Thai subpopulations, three (rs671, rs3782886 and rs7535528) have previously been reported to be associated with disease in the Chinese [41-43]. The documented rapid expansion and assimilation of very recent (within 200 years) Chinese immigrants into Thailand (see Introduction) has thus created a sizeable genetically distinct Sino-Thai subpopulation. Other evidence to support a subpopulation of Sino-Thai includes the presence of an “EAsian” *Helicobacter pylori* haplotype among Thais, which is also found in Malays of recent Chinese descent [51].

The predominantly southern Thai subpopulation SP19/SPA is distinguishable from the other Thai subpopulations by the presence of minor ADMIXTURE-inferred ancestry at K=7 (pink component, Figure 3). This ancestry is a major component of subpopulations SP8-11 comprised of predominantly South and Central Asians. This ancestry in the SP19/SPA Thais may be the signal of earliest Australo-Melanesian ancestors who came from South and Central Asia and migrated via Southeast Asia to Australia. Other genetic evidence of these very early ancestors was reported in [28], who found that the Sakai from southern Thailand were the most diverged ethnic group from other Thais. The Sakai are a very small ethnic group living near the Malaysian border and have a Negrito appearance and speak their own Austroasiatic language similar to Semang Negritos in Malaysia [52]. Among the top-ranked SNP markers which are present at higher frequency in SP-A and distinguish it from the other four Thai subpopulations, two are in genes, namely SLC24A5 and OCA2, known to be associated with skin pigmentation in different populations. However, the association of skin pigmentation with these marker among Asian populations is weak, e.g., as shown among different aboriginal populations of Peninsula Malaysia [53]. The differences in allele frequencies for these markers, and others (Table 2), are thus not likely to reflect signals of selection among Thai subpopulations.

The other Thai subpopulations SP20/SPB and SP21/SPC are the two largest. Among the three SNPs which distinguish SPB from the other Thai subpopulations, one at higher frequency in the HLA-DPB1 gene has been reported to confer a pediatric asthma risk (Table 2). Although the MAF differences among disease associated SNPs appear small among Thai subpopulations, they collectively may nonetheless have important consequences for GWAS. It is well-known that cases and controls must be drawn from a similar genetic background for GWAS, otherwise spurious associations will result [54]. We propose that future GWAS for the Thai population must take into account of the subpopulation background to avoid population structure confounding effects such as spurious associations and loss of power to detect subpopulation-specific disease associations. Regional grouping of samples may not be effective, particularly for the Central region where no one subpopulation is in the majority. 

## Conclusions

This study has elucidated the Thai population structure, revealing four major subpopulations. A major ancestry is common across these subpopulations, which is probably the signal of Austric ancestors who originally settled across most of MSEA. The more recent expansion of Tai-Kadai language throughout MSEA was thus accompanied by assimilation, rather than displacement of the indigenous people. On the other hand, the most recent assimilation of southern Chinese migrants has created shifts in population structure, with one example being the presence of a distinctive Sino-Thai subpopulation that is concentrated in the Central region of Thailand (but which is not in the majority). 

Further sampling of genetic variation in other MSEA populations, particularly Vietnamese and Cambodians may shed further light on this pattern. 

## Supporting Information

File S1 **List of SNP-ids for the 41,569 SNP markers common to the Illumina Human 610-Quad BeadChips Array and the Affymetrix Human SNP Array 6.0 platforms.**
(ZIP)

Figure S1 **MAF correlation of 41,569 SNPs between Illumina and Affymetrix platforms.** MAFs for each SNP were calculated from a control population of European ancestry with 136 samples from Affymetrix [29] and 1,182 samples from Illumina [31] platforms, respectively. The calculated correlation coefficient is indicated by the red line.
(TIFF)

Figure S2 **ipPCA clustering decision tree for analysis of combined datasets 1, 2 and 3 (worldwide datasets).** The terminal nodes boxed in red represent ipPCA resolved subpopulations labeled SP1-24. The internal nodes represent groups of individuals with unresolved population structure. Terminal nodes marked with asterisks represent outlier individuals. The EigenDev value for each iteration of ipPCA is shown in each node; values >0.21 indicate the present of substructure.
(PDF)

Figure S3 **ipPCA clustering decision tree for analysis of combined datasets 2 and 3 (Thai individuals).** The terminal nodes boxed in red and labeled as SPA, SPB, SPC, and SPD represent ipPCA resolved subpopulations. Terminal nodes marked with asterisks represent outlier individuals. The numbers of individuals for each regional origin label (Thai C, S, NE and N) are indicated in each node. The intermediate nodes represent groups of individuals with unresolved population structure. The EigenDev value for each iteration of ipPCA is shown in each node; values >0.21 indicate the present of substructure.
(TIFF)

Table S1 **Major depressive disorder GWAS top 50 associated SNP data.**
(XLSX)

Table S2 **Correspondence of individual ipPCA-assignments of SP19-22 with SPA-D.**
(XLSX)

Table S3 **Top 50 rank SNP from Chi-squared analysis between Thalassemia dataset and the Major depressive disorder dataset from the expected ratio for all markers.**
(XLSX)

Table S4 **Top 200 ranked SNPs based on Fst values for all pair-wise comparisons between SPA, SPB, SPC and SPD.**
(XLSX)
