# Supplementary figures and images for: Insight into the Peopling of Mainland Southeast Asia from Thai Population Genetic Structure
Source: PLoS One. 2013 Nov 4;8(11):e79522. doi: 10.1371/journal.pone.0079522 (PMC3817124; doi:10.1371/journal.pone.0079522)

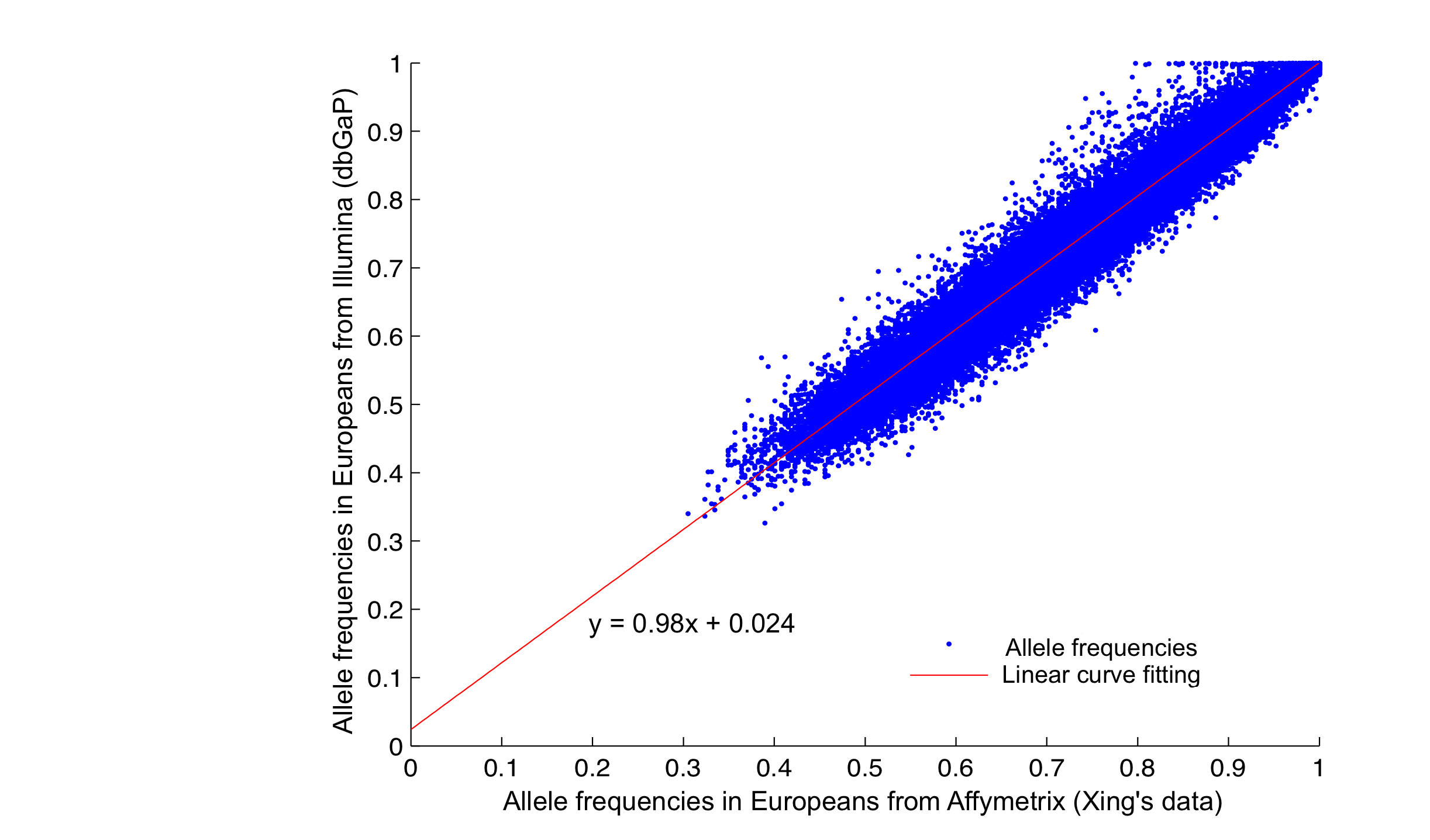

Supplement: Figure S1 — MAF correlation of 41,569 SNPs between Illumina and Affymetrix platforms. MAFs for each SNP were calculated from a control population of European ancestry with 136 samples from Affymetrix [29] and 1,182 samples from Illumina [31] platforms, respectively. The calculated correlation coefficient is indicated by the red line. (TIFF) [file pone.0079522.s002.tiff]

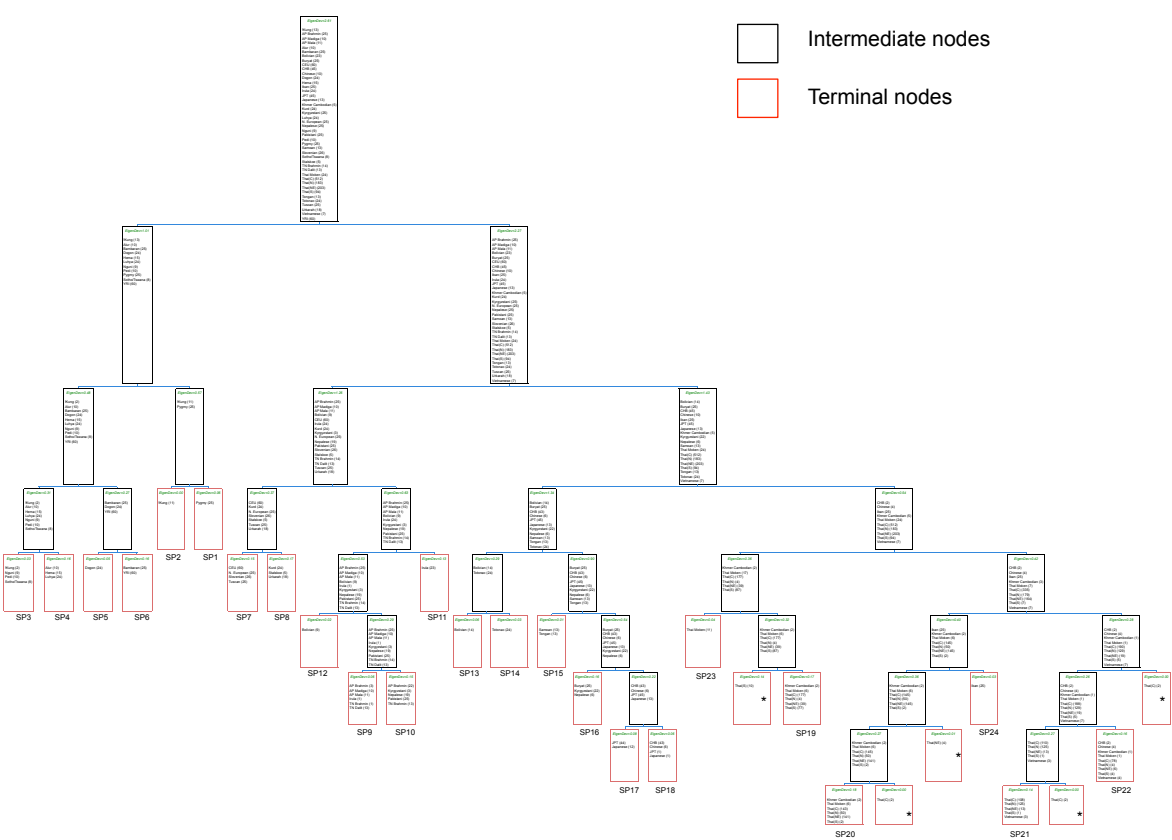

Supplement: Figure S2 — ipPCA clustering decision tree for analysis of combined datasets 1, 2 and 3 (worldwide datasets). The terminal nodes boxed in red represent ipPCA resolved subpopulations labeled SP1-24. The internal nodes represent groups of individuals with unresolved population structure. Terminal nodes marked with asterisks represent outlier individuals. The EigenDev value for each iteration of ipPCA is shown in each node; values >0.21 indicate the present of substructure. (PDF) [file pone.0079522.s003.pdf]

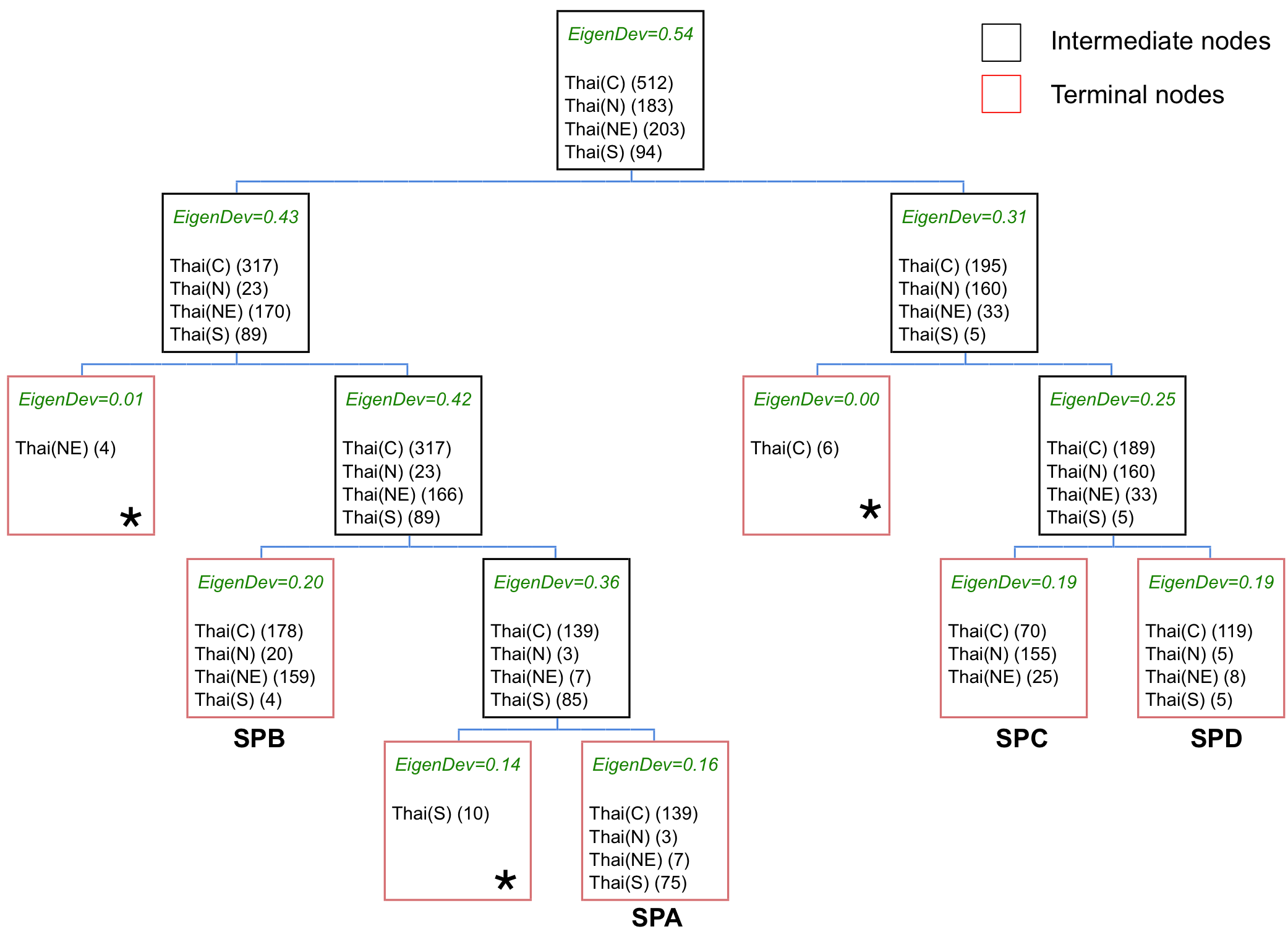

Supplement: Figure S3 — ipPCA clustering decision tree for analysis of combined datasets 2 and 3 (Thai individuals). The terminal nodes boxed in red and labeled as SPA, SPB, SPC, and SPD represent ipPCA resolved subpopulations. Terminal nodes marked with asterisks represent outlier individuals. The numbers of individuals for each regional origin label (Thai C, S, NE and N) are indicated in each node. The intermediate nodes represent groups of individuals with unresolved population structure. The EigenDev value for each iteration of ipPCA is shown in each node; values >0.21 indicate the present of substructure. (TIFF) [file pone.0079522.s004.tiff]
